# Clinical translation of mesenchymal stromal cell extracellular vesicles: Considerations on scientific rationale and production requisites

**DOI:** 10.1111/jcmm.17112

**Published:** 2021-12-20

**Authors:** Yvan Courageux, Marta Monguió‐Tortajada, Cristina Prat‐Vidal, Antoni Bayes‐Genis, Santiago Roura

**Affiliations:** ^1^ ICREC Research Program Health Science Research Institute Germans Trias i Pujol Badalona Spain; ^2^ Heart Institute (iCor) Germans Trias i Pujol University Hospital Badalona Spain; ^3^ CIBERCV Instituto de Salud Carlos III Madrid Spain; ^4^ Servei de Teràpia Cel·lular Banc de Sang i Teixits Barcelona Spain; ^5^ Cardiology Service Germans Trias i Pujol University Hospital Badalona Spain; ^6^ Department of Medicine Universitat Autonoma de Barcelona Barcelona Spain; ^7^ Institut d’Investigació Biomèdica de Bellvitge‐IDIBELL L´Hospitalet de Llobregat Spain; ^8^ Faculty of Medicine University of Vic‐Central University of Catalonia Vic Spain

**Keywords:** biomanufacturing, clinical translation, extracellular vesicles, mesenchymal stromal cells, size exclusion chromatography

## Abstract

The present paper is a commentary to *‘Identification and characterization of hADSC*‐*derived exosome proteins from different isolation methods*’ (Huang *et al*. 2021; 10.1111/jcmm.16775). Given the enthusiasm for the potential of mesenchymal stromal cell‐derived extracellular vesicles (MSC‐EVs), some considerations deserve attention as they move through successive stages of research and application into humans. We herein remark the prerequisite of generating that evidence ensuring a high consistency in safety, composition and biological activity of the intended MSC‐EV preparations, and the suitability of disparate isolation techniques to produce efficacious EV preparations and fulfil requirements for standardized clinical‐grade biomanufacturing.

Available preclinical findings indicate that mesenchymal stromal cell‐derived extracellular vesicles (MSC‐EVs) are highly versatile and powerful biologicals in terms of regenerative and anti‐inflammatory properties for the development of efficient cell‐free therapies.^1^ Data point out that MSC‐EVs trigger effects that are comparable to those of their parental cells. Also, they display better stability and handling as well as reduced toxicity with no alloreactivity, while not being associated with tumorigenic nor thrombogenic risk after intravascular administration, in addition of having a targetable biodistribution. Thus, much effort is currently focused on their effective clinical application. In this context, Huang et al. characterized the differences in protein cargo from MSC‐EVs after using ultracentrifugation (UC), size exclusion chromatography (SEC), ExoQuick‐TC precipitation and ExoQuick‐TC ULTRA as isolation methods.^2^ In our opinion, the presented results lead to some crucial questions.

On the question ‘Do we have enough evidence of the therapeutic value of MSC‐EVs to endorse their clinical application?’, many animal experimental studies in diverse disease in vivo models suggest that MSC‐EV administration is safe and promotes beneficial effects.^3^ However, despite the efforts for standardized characterization agreed with International Society for Extracellular Vesicles recommendations,^4^ the lack of consensus in a common EV isolation strategy together with the frequent coarse description of EV preparations and the discrepancies in doses used in disease models make it difficult to compare data from overly heterogeneous EV preparations.^5^ This leads to a non‐negligible bias as well as noteworthy limitations and difficulties in extrapolating results from a plethora of scientific literature on the potential of MSC‐EVs. For instance, Huang et al. highlight the different compositions of EV samples depending on the type of isolation technique used, as others have highlighted.^6^ At the same time, although Huang et al. used electron microscopy to characterize the obtained preparations, higher settings for both image size and quality are more recommendable to be applied to undoubtedly discriminate whole double‐membrane EVs from non‐intact vesicles, highly electron‐dense non‐vesicular particles or simply protein aggregates to evaluate EV integrity and presence of gross impurities. In parallel, the use of Nanoparticle Tracking Analysis for establishing the yield/recovery of EVs comparing different isolation techniques has a limited reliability, as while being one of the currently available EV quantification tools it can overestimate the number of particles when EV preparations are not pure enough. These are just examples of the issues that need a rigorous evaluation to ensure trustworthy conclusions and continue pursuing the clinical application of MSC‐EV products. Therefore, the scientific community, working back‐to‐back with official regulatory agencies, should focus on generating data from robust and well‐characterized MSC‐EV samples, with safety and efficacy at the centre of the research, and anticipating relevant experiments and models to retrieve clues on the mechanisms of action.^7^

Given today's different in‐house EV separation strategies that result in varied degrees of yield, purity and composition as aforementioned, the second question is as follows: ‘Which of the technical procedures shows the greatest applicability and ability to adapt to large‐scale production while meeting current good manufacturing practices and regulatory instructions?’. We and others agree with the authors that the diversity in molecular contents specifically reflects the wide range of biological functions of isolated MSC‐EVs, as well as certain advantages and disadvantages of each method's impact on forthcoming uses, preferentially those aimed at patients.^8^ Regarding the methods that Huang et al. analysed, UC has generally been considered as the most conventional method for MSC‐EV isolation, from either cells or biological fluids, but it is relatively time‐consuming and requires large sample volume processing and access to specialized laboratory equipment that are not always available to clinical researchers. Furthermore, UC has been demonstrated to affect integrity of EVs, and pellet EVs together with protein aggregates and other impurities present in the starting material. Thus, it is difficult to discern if the resulting biological activity is predominantly associated with EVs instead of with co‐isolated soluble mediators. The same happens with alternative approaches based on adding precipitating agents such as ExoQuick‐TC, which more quickly and easily recover EVs from low‐volume samples than UC, but also with other non‐EV impurities. Moreover, there are some techniques allowing a stringent EV separation, either based on gradient centrifugations or immunoaffinity isolation, which allow distinction of subpopulations but would be not suited at all for large‐scale production due to either their intrinsic workflow or high time‐consuming and costs. SEC, an isolation methodology based on size separation using prepacked chromatography columns, is superior in isolating well‐defined EVs achieving effects that resemble those of parental MSCs.^9^ This technique is cost‐efficient, greatly reducing the amount of impurities in the EV preparation, and it can be easily combined with tangential flow filtration for clarification and upstream/downstream concentration to obtain pure EVs from large‐scale productions. While it has been described that serum‐derived lipoproteins may remain,^10^ there are approximations combining SEC with anionic affinity isolation or using different sizes of SEC matrix if they interfere with EV function and thus their presence needs to be avoided. To achieve clinical translation, in addition to meeting the aimed scale range, production must necessarily warrant high reproducibility as well as an unequivocal safety profile associated with therapeutic benefit. In this sense, the implementation of a scalable MSC‐EV production is essential. To that end, growing attention is also given to the use of bioreactors and chemically defined medium which promise scalability, better reproducibility and less safety concerns. Additionally, specific preconditioning of parental MSCs could stimulate the production of EVs displaying modified cargoes and enhanced therapeutic potential.

## CONFLICTS OF INTEREST

The authors confirm that there are no conflicts of interest.

## AUTHOR CONTRIBUTION

**Yvan Courageux:** Writing – original draft (equal); Writing – review & editing (equal). **Marta Monguió‐Tortajada:** Writing – review & editing (equal). **Cristina Prat‐Vidal:** Writing – review & editing (equal). **Antoni Bayes‐Genis:** Funding acquisition (equal); Writing – review & editing (equal). **Santiago Roura:** Conceptualization (lead); Funding acquisition (lead); Writing – original draft (equal); Writing – review & editing (equal).

## Data Availability

Data sharing not applicable – no new data generated, or the article describes entirely theoretical research
